# Regulatory Role of lncRNA *MEG3* Silencing on *PI3K/GSK3β/Tau* Pathway in a High-Glucose-Induced Cell Model

**DOI:** 10.3390/ijms26167944

**Published:** 2025-08-18

**Authors:** Lütfiye Ozpak

**Affiliations:** Department of Medical Biology, Faculty of Medicine, Sütçü İmam University, Kahramanmaraş 46040, Turkey; lutfiyeozpak@gmail.com

**Keywords:** Alzheimer’s disease, long non-coding RNA, *MEG3*, high glucose, neurotoxicity

## Abstract

This study investigated the regulatory role of the long non-coding RNA maternally expressed gene 3 (*MEG3*) in tau hyperphosphorylation and insulin signaling (*PI3K/AKT1/GSK3β*) under high-glucose (HG)-induced neurotoxic conditions mimicking Alzheimer’s disease pathology. To explore the function of *MEG3* within a hyperglycemic (Hyp) model, *MEG3* was silenced using small interfering RNA (siRNA) assay, followed by Western blot analysis, qRT-PCR, and network analyses. The si*MEG3* + Hyp group had lower levels of *AKT1* (0.48-fold) and *PI3K* (0.52-fold) than did the Hyp group. In the si*MEG3* + Hyp group, *GSK3β* (2.51-fold) and *TNFα* (2.38-fold) expressions were higher than were those in the Hyp group, while in the si*MEG3* group, GSK3β (4.59-fold), microtubule-associated protein *TAU* (*MAPT*, *TAU*) (6.37-fold), interleukin *(IL)1β* (5.67-fold), *IL6* (3.29-fold), and tumor necrosis factor-α (*TNFα*) (3.06-fold) were all significantly upregulated in comparison to the control group. A higher level of p-tau protein was seen in the si*MEG3* group in comparison to the control group, as well as in the si*MEG3* + Hyp group in comparison to the Hyp group. Gene ontology analysis following *MEG3* administration showed that genes downstream of the *PI3K* pathway were suppressed, whereas genes regulating the neuroinflammatory response were upregulated. The results suggest that the lncRNA *MEG3* may be a promising therapeutic target in HG-induced neurodegenerative AD.

## 1. Introduction

Alzheimer’s disease (AD) is a gradually worsening neurodegenerative disease whose primary causes are atrophy and loss of neurons in the cerebral cortex. Histologically, it is characterized by β-amyloid plaques and neurofibrillary tangles resulting from tau hyperphosphorylation [1]. The 2023 Alzheimer’s Disease Facts and Figures Report shows that 6.7 million Americans over the age of 65 suffer from Alzheimer’s type dementia, and by 2060, this figure is predicted to rise to 13.8 million, with an additional 200,000 individuals experiencing early-onset dementia, if nothing is done [2]. Type 2 diabetes (T2D) and AD share many common pathological mechanisms, primarily insulin resistance [3]. Recognized as a type of diabetes and referred to as “Type 3 diabetes” (T3D), AD occurs through impaired insulin signaling in the hippocampus and the concurrent development of insulin resistance [4,5]. Approximately 75% of individuals with AD have been identified with T2D and glucose intolerance [6]. Additionally, the literature indicates that chronic hyperglycemia impacts hippocampal neurogenesis and the brain’s ability to handle AD-related pathological burdens, resulting in impaired learning and memory-processing decline [7]. Hyperglycemia exposure and insulin resistance trigger neuroinflammation by causing damage to both neuronal and non-neuronal cells through mechanisms such as microglial activation, oxidative stress, and decreased Aβ elimination [8]. Studies conducted both in vitro and in vivo have demonstrated that insulin resistance may contribute to Alzheimer’s pathogenesis through various pathways [9,10]. Insulin-*PI3K/AKT* signaling cascade components in the brain preserve synaptic plasticity in memory [11]. Chronic exposure to HG levels leads to the disruption of *PI3K/AKT* signaling homeostasis, increased activity of *GSK3β*—a downstream molecule of *AKT* that regulates glycogen synthesis in response to insulin signaling—and consequently, increased tau hyperphosphorylation and Aβ deposition. Accumulation of Aβ can compete with insulin for binding to the insulin receptor, thereby reducing the activity of the *PI3K/AKT* pathway [12,13]. Regulatory RNAs are essential for controlling intricate processes such as memory, learning, and cognitive function. Dysregulation of *MEG3* lncRNA expression in Alzheimer’s disease plays a critical role in disease progression by influencing necrosis and apoptosis processes in neuronal cells; recent studies have further identified *MEG3* as a key regulator of neuronal necroptosis, highlighting its potential role in human-specific neuronal vulnerability and offering novel therapeutic targets [14,15]. *MEG3* is a regulatory long non-coding RNA that is important in Alzheimer’s pathology due to its relationship with *PI3K/AKT* and tau hyperphosphorylation [16].

No study in the literature has investigated the effect of lncRNA *MEG3* on the *PI3K/GSK3β/TAU* signaling pathway in hyperglycemic neurotoxic models in vitro and in vivo. The lncRNA, *MEG3*, which regulates the insulin signaling pathway implicated in the molecular mechanism of T3D, may be a molecular therapeutic target for managing the onset of complications and the advancement of the disease brought on by diabetes and Alzheimer’s disease. This study determined the effect of regulatory lncRNA *MEG3* on tau hyperphosphorylation level and *PI3K/AKT1/GSK3β* insulin signaling pathway, which plays a role in Alzheimer’s pathology, in the human neuroblastoma SH-SY5Y cell line by inducing neurotoxicity through stimulation with HG.

## 2. Results

### 2.1. High Doses of Glucose Induced Neurotoxicity on the Viability of SH-SY5Y Cells

**Figure 1:**
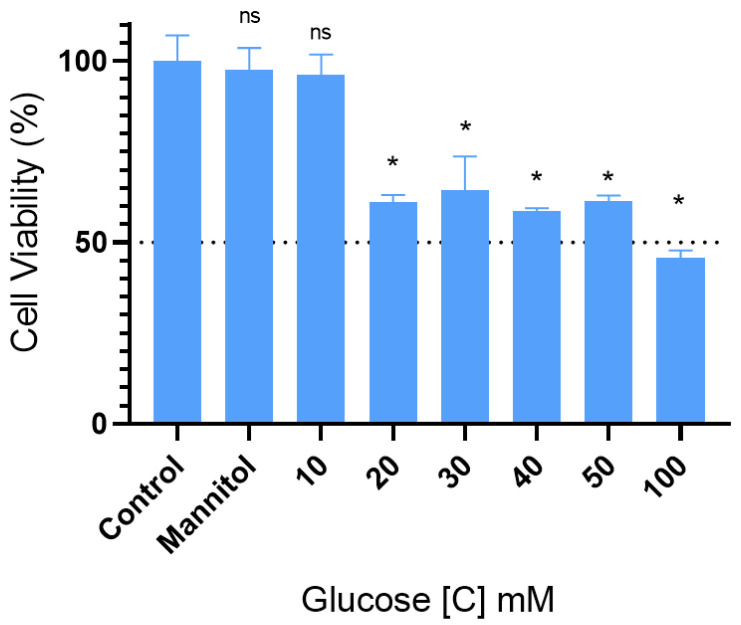
Following a 24-h incubation period, the MTT assay was performed to determine the viability of SH-SY5Y cells, illustrating the impact of progressively raising the glucose content. Data are presented as mean ± SEM; *n* = 3. (ns) indicates no significant difference; (*) *p* < 0.001 denotes a significant difference compared to 100 mM of glucose. Additionally, cell viability decreased to below 50% compared to the control group.

Using an MTT test, we evaluated the influence of various glucose treatment doses on neurotoxicity in SH-SY5Y cells. SH-SY5Y cell viability was assessed following treatment with increasing concentrations of glucose (10, 20, 30, 40, 50, and 100 mM). A 100 mM glucose dose reduced cell viability to 45.66 ± 2.16% (*p* < 0.001) (Figure 1). Therefore, 100 mM of high glucose (HG) was chosen as a neurotoxic dose to develop the SH-SY5Y cell damage model for further investigations. Mannitol was used as an osmotic control to separate the effects of high glucose from those caused by increased osmolarity. No significant difference was observed between the group treated with mannitol and the normal control group, indicating that the observed cellular effects were specific to high glucose and not related to osmotic stress.

### 2.2. MEG3 siRNA Transfection Altered Insulin Signaling Pathway-Related Gene Expression in Glycemic and Hyperglycemic Model SH-SY5Y Cells

**Figure 2:**
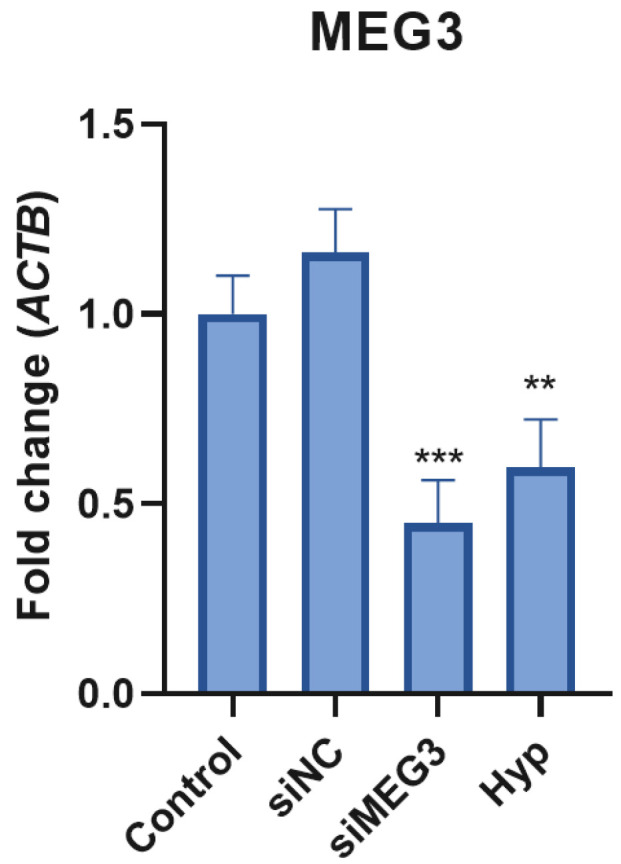
lncRNA *MEG3* expression levels were measured under different conditions, including control, siNC, si*MEG3*, and hyperglycemia (Hyp). Both the si*MEG3* and Hyp groups showed a significant decrease in *MEG3* expression compared to the control group (*p* < 0.001 (***) and *p* < 0.05 (**), respectively).

After transfection, real-time PCR was conducted to ascertain the biological role of lncRNA *MEG3* in a neurotoxic environment induced by HG, which is identical to the T3D condition. The expression of si*MEG3* and hyperglycemia groups was considerably less than that of the control groups (*p* < 0.001 and *p* < 0.05, respectively), as illustrated in Figure 2.

**Figure 3:**
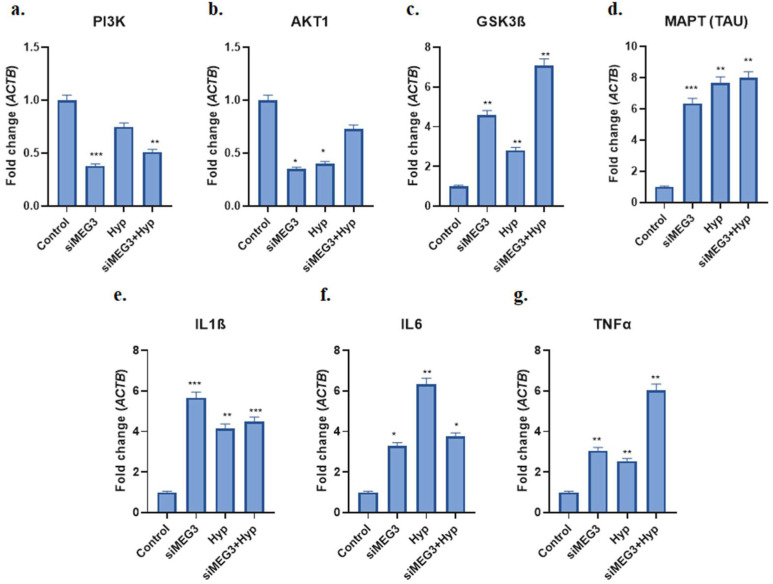
The impact of si*MEG3* transfection on *PI3K*, *AKT1*, *GSK3β*, *TAU*, *IL1β*, *IL6*, and *TNFα* mRNA expression levels in a neurotoxic environment triggered by HG that is similar to the T3D state. mRNA expressions of *PI3K* (**a**), *AKT1* (**b**), *GSK3β* (**c**), *MAPT (TAU)* (**d**), *IL1β* (**e**), *IL6* (**f**), and *TNFα* (**g**) were determined by real-time PCR after si*MEG3* transfection. Fold changes were normalized to β-actin. The data are the mean ± standard deviation of three separate independent experiments. Statistical analysis was conducted using one-way ANOVA followed by Tukey’s test, and *p* < 0.05 was considered statistically significant. (* *p* < 0.05, ** *p* < 0.01, *** *p* < 0.001).

Figure 3 displays the relative ratio of the groups’ changes in *PI3K*, *AKT1*, *GSK3β*, *TAU*, *IL1β*, *IL6*, and *TNFα* mRNA expressions. *PI3K* and *AKT1* gene expression levels were considerably reduced in the lncRNA *MEG3* knockdown (si*MEG3*) group as compared to the other groups. Compared to the control and hyperglycemia groups, a decrease in the expression level of the *PI3K* gene was observed in the si*MEG3* + Hyp group. The si*MEG3* group had considerably lower expressions of *PI3K* (0.38-fold; *p* < 0.001) and *AKT1* (0.35-fold; *p* < 0.01) than did the control group. In comparison to the Hyp group, the si*MEG3* + Hyp group had an elevated *AKT1* level (0.48-fold, *p* < 0.05) and a lower *PI3K* (0.52-fold, *p* < 0.01) expression (Figure 3a,b).

*GSK3β*, *TAU*, *IL1β*, *IL6*, and *TNFα* gene expression levels were significantly elevated (4.59-, 6.37-, 5.67-, 3.29-, and 3.06-fold; *p* < 0.05, respectively) in the si*MEG3* group in comparison with the control group, demonstrating that these molecules have the strongest connections with Alzheimer’s pathogenesis. *GSK3β* and *TNFα* gene expression levels were observed to be elevated (2.51- and 2.38-fold; *p* < 0.05, respectively) in the si*MEG3* + Hyp group as compared to the Hyp group (Figure 3c–g).

### 2.3. Knockdown of lncRNA MEG3 in Hyperglycemic SH-SY5Y Cells Mimicked Alzheimer’s Pathology Through Increased p-Tau Protein Level

**Figure 4:**
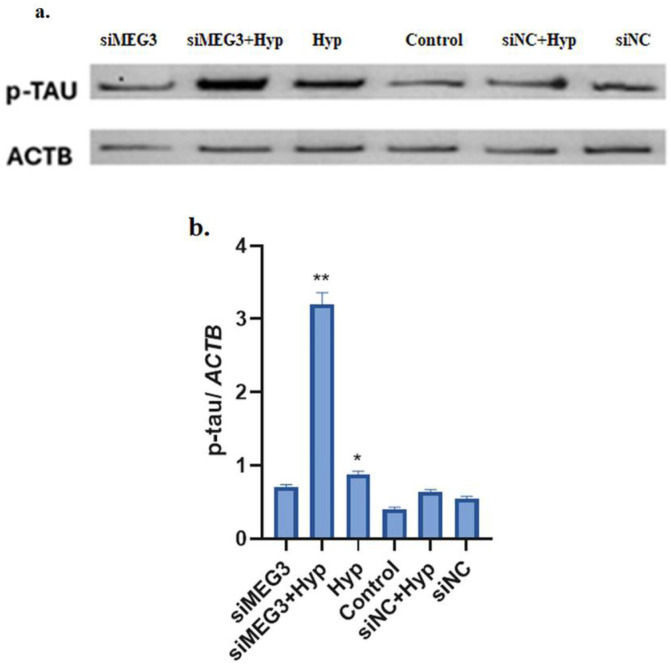
The expression levels of p-tau (**a**) protein were evaluated by Western blot analysis using ImageJ software. siRNA was used to silence the lncRNA *MEG3*, and changes in these proteins were examined in hyperglycemic and normoglycemic cell models. ACTB was used as the internal control. Histogram displaying the densitometric analysis result of p-tau (**b**) protein level was normalized to the control group. (*MEG3*: maternally expressed gene 3; si*MEG3*: small interfering RNA maternally expressed gene 3; siNC: negative control siRNA; Hyp: hyperglycemic; *ACTB*: beta-actin; p-tau: phosphorylated tau; (*) *p* ≤ 0.05 indicates a significant difference compared to control; (**) *p* ≤ 0.001 indicates a significant difference comparison to control.

Western blot results confirmed that the increased pathological phosphorylation of p-tau may contribute to the progression of tauopathies (Figure 4).

### 2.4. MEG3 Was Linked to GSK3β and Regulated PI3K Signaling and Neuroinflammation

**Figure 5:**
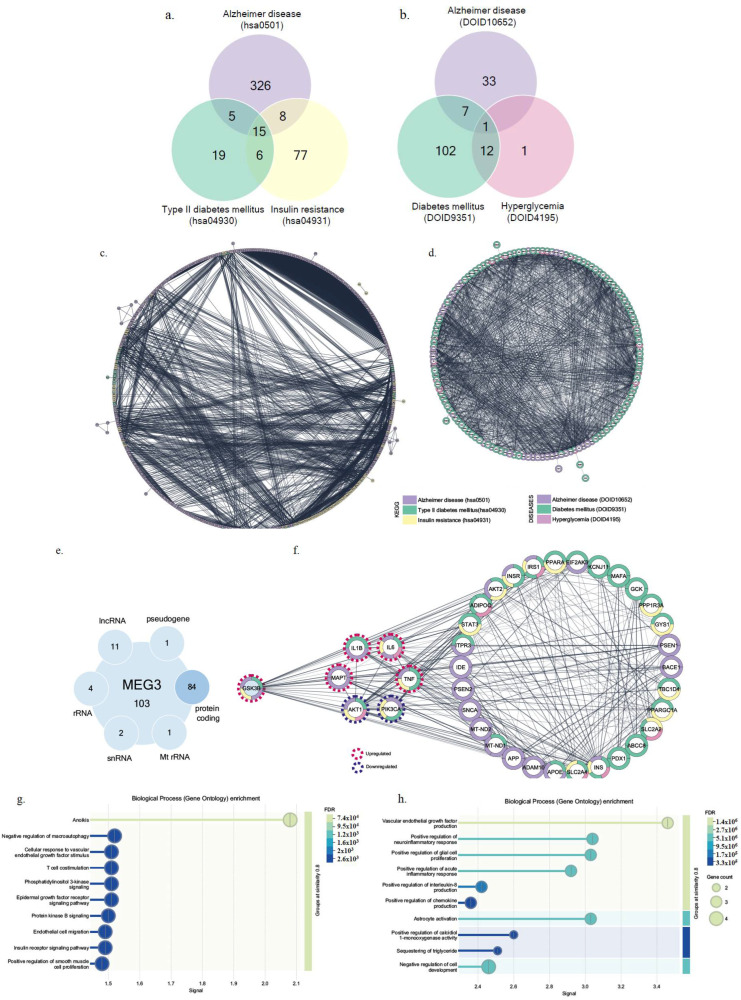
(**a**) Venn diagram showing the overlap of genes identified through KEGG pathway enrichment analysis for Alzheimer’s disease (hsa05010), type II diabetes mellitus (hsa04930), and insulin resistance (hsa04931). The numbers represent the genes uniquely associated with each condition as well as those shared among them. (**b**) Venn diagram illustrating the overlap of genes identified through disease–gene association (DISEASES) enrichment analysis for Alzheimer’s disease (DOID:10652), diabetes mellitus (DOID:9351), and hyperglycemia (DOID:4195). The numbers indicate the genes uniquely or commonly associated with each condition. (**c**,**d**) Graphs of network analysis of enriched terms in the mentioned pathways. Purple represents Alzheimer’s disease (hsa05010 and DOID:10652), green represents diabetes mellitus (hsa04930 and DOID:9351), yellow represents insulin resistance (hsa04931), and pink represents hyperglycemia (DOID:4195). (**e**) Representation of the types of RNA molecules associated with *MEG3* (ENSG00000214548) and the numbers representing each RNA type. GSK3β, which is among the protein-coding targets of *MEG3* lncRNA, is also included. (**f**) Network analysis of genes found in both the KEGG and DISEASES categories is shown. Among these genes, *GSK3β* was also included in the *MEG3*-associated genes, and it was determined that its mRNA level was upregulated in the groups where *MEG3* was silenced. In addition to *GSK3β*, upregulation of the *MAPT (TAU)*, *IL1β*, *IL6*, *and TNFα* genes was also observed (indicated by red dashed lines). These treatments led to the downregulation of *AKT1* and *PIK3CA* expression levels (indicated by blue dashed lines). (**g**) Gene Ontology (GO) biological process enrichment analysis of downregulated genes following *MEG3* application. The analysis revealed significant enrichment in phosphatidylinositol 3-kinase signaling and other related pathways, with false-discovery rates (FDR) and gene counts indicated. (**h**) Gene Ontology (GO) biological process enrichment analysis of upregulated genes following *MEG3* application. The results show significant enrichment in positive regulation of neuroinflammatory response and other inflammation-related pathways, with FDR and gene counts displayed.

KEGG pathway enrichment analysis identified 354, 45, and 106 genes associated with Alzheimer’s disease (term ID: hsa05010), type II diabetes mellitus (term ID: hsa04930), and insulin resistance (term ID: hsa04931), respectively (Figure 5a). Among these, *IKBKB*, *INS*, *INSR*, *IRS1*, *IRS2*, *MAPK8*, *MAPK9*, *MTOR*, *PIK3CA*, *PIK3CB*, *PIK3CD*, *PIK3R1*, *PIK3R2*, *PIK3R3*, and *TNF* were common to all three pathways (Figure 5c).

Similarly, disease–gene association (DISEASES) enrichment analysis identified 41, 122, and 14 genes for Alzheimer’s disease (term ID: DOID:10652), diabetes mellitus (term ID: DOID:9351), and hyperglycemia (term ID: DOID:4195), respectively (Figure 5b). IL6 was identified as a common gene in all three conditions (Figure 5d). The identified genes and their associated pathways are listed in Supplementary Table S2. A total of 102 RNA molecules associated with *MEG3* (ENSG00000214548) were identified (Figure 5e, Supplementary Table S3). When these RNAs were analyzed in relation to the 36 genes enriched in both KEGG and DISEASES categories, *GSK-3β* was found to be the only gene also associated with *MEG3* (Figure 5f).

Biological process (Gene Ontology) enrichment analysis of genes differentially expressed following *MEG3* application revealed that downregulated genes were involved in phosphatidylinositol 3-kinase signaling (Figure 5g), whereas upregulated genes were associated with positive regulation of neuroinflammatory response (Figure 5h).

## 3. Discussion

lncRNAs stand out as promising biomarkers and therapeutic targets in AD in serum or cerebrospinal fluid (CSF) owing to their stable structure [17]. Despite thorough research on the function of the lncRNA *MEG3* in many forms of cancer, there are still few studies examining its role in the relationship between AD and T2D. This study underlines *MEG3*’s potential role in these concatenate diseases by being the first to examine its impact on the *PI3K/AKT/GSK3β* signaling pathway in a hyperglycemic cell culture. The findings demonstrated that SH-SY5Y neuronal cells exposed to high glucose concentrations markedly reduced *PI3* and *AKT1* levels but increased those of *GSK3β* and Tau. This indicates that our hyperglycemia cell model exhibits impaired insulin signaling, consistent with findings from diabetic models in previous studies [18,19].

Numerous studies have shown that chronic exposure to hyperglycemia is closely associated with permanent cognitive impairment [20]. It is known that cognitive impairments associated with hyperglycemia arise through various mechanisms such as oxidative stress, inflammation, apoptosis, and ROS production in hippocampal neurons responsible for learning and memory [21]. In a study conducted on primary hippocampal neurons, it was demonstrated that overexpression of lncRNA *MEG3* alleviated mitochondrial dysfunction, neuronal toxicity, and apoptosis caused by high glucose levels and that *MEG3* can translocate to the mitochondria, although the mechanism of this translocation has not yet been clarified [22]. In this context, the increase in the expression of inflammation related markers observed in this study with the silencing of *MEG3* under conditions of neurotoxicity caused by high glucose supports the role of lncRNA *MEG3* in both protecting mitochondrial functions and suppressing the inflammatory response.

By interfering with glucose uptake in cells through abnormalities in the insulin signaling pathway, hyperglycemia causes insulin resistance. Hence, the *PI3K/AKT1* signaling cascade is deactivated, which is crucial for maintaining brain homeostasis and cognitive function. This degraded state activates *GSK3β*, leading to hyperphosphorylation of Tau protein and the release of pro-inflammatory cytokines with increased neurotoxicity; biomarkers such as advanced glycation end products (AGEs), *IRS1*, *TNFα*, hs-CRP, leptin, *IL1β*, and *IL6* suggest potential diabetes-related cognitive impairment [23,24]. Our results also showed that hyperglycemic SH-SY5Y model neuronal cells exhibited a marked decrease in *PI3K* and *AKT1* levels and a significant increase in *GSK3β* activation and expression of tau, *IL1β*, *IL6*, and *TNFα*. This supports the hypothesis that hyperglycemia-induced impairment in insulin signaling exacerbates neuroinflammation and tau pathology and is in line with previous studies showing a link between metabolic dysfunction and cognitive impairment in diabetes.

lncRNAs influence AD pathogenesis and disease process by altering biochemical and metabolic pathways such as transcription and post-translational regulation, chromatin modification, and organization of protein complexes. In this context, due to its positive regulatory role, a decrease in the level of lncRNA *MEG3* may contribute to AD progression, which is closely linked to T3D, by affecting brain insulin resistance. Upregulation of *MEG3*, by regulating insulin signaling in the brain, may offer neuroprotective effects and potentially alleviate cognitive impairment and neuronal damage associated with AD pathogenesis [25]. A previous investigation revealed that upregulating the lncRNA *MEG3* may reverse cognitive decline and lessen neuronal damage. Furthermore, deactivating the *PI3K/AKT* signaling pathway in AD causes the hippocampus’s astrocytes to become inactive, which makes *MEG3* an appealing biomarker and treatment target for AD [16]. Furthermore, this study’s findings align with our results showing that silencing of lncRNA *MEG3* causes inactivation of the *PI3K/AKT1* signaling pathway. Previous research has shown that the *PI3K/AKT/GSK3β* signaling pathway is critical in neuroinflammation and neurodegenerative diseases, particularly AD [26]. In this context, it has been reported that the accumulation of Aβ oligomers, one of the earliest pathological features of Alzheimer’s disease, removes insulin receptors from the neuronal surface and directly inhibits the *PI3K/Akt* signaling pathway, thereby preventing its activation. As a result, the activity of the *GSK3β* enzyme increases, leading to abnormal hyperphosphorylation of the Tau protein and accelerating the progression of the disease toward neurodegeneration [27]. A previous study reported that icariin (ICA) alleviated Alzheimer’s disease pathology by regulating the expression of *MEG3* and *MALAT1* lncRNAs and modulating the *AKT/GSK3β* signaling pathway [28]. In our in vitro study using a hyperglycemic neurotoxic model in which *MEG3* was silenced, an increase in *IL1β*, *IL6*, and *TNFα* levels, which are markers of neuroinflammation, was also observed. It has been discovered that lncRNA *MEG3* is crucial for many biological activities, including inflammation and microglial activation [29]. Overexpression of *MEG3* was observed to improve spatial learning and memory skills in AD rats. *MEG3* attenuates inflammation in the hippocampus of AD rats by reducing Aβ 25-35 accumulation and oxidative damage and decreasing *IL1β*, *IL6*, and *TNFα* levels. Furthermore, overexpression of *MEG3* suppresses astrocyte activation in the hippocampus of AD patients by inhibiting the *PI3K/AKT* signaling pathway. Moreover, it has been reported that *MEG3* reduces oxidative stress and inflammatory damage and promotes neuronal survival by inhibiting pathological damage of hippocampus neurons, which may be related to posttranscriptional modulation of gene expression [16,30]. In this study, it was observed that the pathological damage associated with hyperglycemia and suppression of lncRNA *MEG3* increased the inflammatory response and decreased the expression levels of genes involved in the *PI3K/AKT1* pathway.

While the mRNA expression changes found in this study do not prove causality on their own, these findings support their importance in AD-related processes, particularly in the context of neuroinflammation and tau pathology. Although this study is limited to in vitro analyses and cannot fully reflect in vivo complexity, our multi-level molecular approaches, including qPCR, Western blot, enrichment, and network analyses, provide preliminary yet valuable insights into the potential regulatory role of *MEG3*. However, the use of a single in vitro neuronal model and the lack of experimental validation for all predicted signaling pathways from in silico analyses are important limitations of this study. The lack of gain-of-function experiments, such as *MEG3* overexpression, is another limiting factor for this study in terms of understanding the dual role of *MEG3*. Although it is frequently preferred in in vitro chronic hyperglycemia models in the literature, the use of concentrations above in vivo levels is a limiting factor in this study. Future studies using in vivo models and high-throughput techniques are needed to confirm and extend these findings.

## 4. Materials and Methods

### 4.1. Materials

Dulbecco’s modified eagle’s medium (DMEM)/low glucose, fetal bovine serum (FBS), and reduced serum medium (Opti-MEM) were purchased from Gibco (Grand Island, NY, USA). D-glucose, dimethyl sulfoxide (DMSO), and phosphate-buffered saline (PBS) were obtained from Sigma-Aldrich (St. Louis, MO, USA). Thiazolyl Blue Tetrazolium Bromide (MTT) was supplied from Gold Biotechnology, St. Louis, MO, USA. siRNA was specific for *MEG3* (Cat#: 4392420), and its non-targeting control as a negative control for *MEG3* (Cat#: 4390843) was purchased from Ambion™ Silencer™ Select Pre-Designed siRNA (Fisher Scientific, Loughborough, UK). Total RNA was isolated using TRIzol™ Reagent (Thermo Fisher Scientific, Waltham, MA, USA), cDNA was synthesized with the High-Capacity cDNA Reverse Transcription Kit (Applied Biosystems, Foster City, CA, USA), and qRT-PCR was performed using the QuantiTect SYBR^®^ Green PCR Kit (Qiagen, Hilden, Germany). Inhibitors were purchased from Roche Diagnostics (Complete Mini Protease Inhibitor Cocktail, Basel, Switzerland) and PhosSTOP Phosphatase Inhibitor Cocktail (Roche Diagnostics, Basel, Switzerland). RNAiMAX transfection reagent (Invitrogen, Carlsbad, CA, USA) used in transfection was donated from Assoc. Prof. Dr. Bahadır Batar (Tekirdağ Namık Kemal University).

### 4.2. SH-SY5Y Cell Culture and High Glucose Treatment

Fewer than 100 passes of the SH-SY5Y human neuroblastoma cell line, which was supplied by Prof. Dr. Gizem Dönmez Yalcın of Adnan Menderes University in Turkey, were employed. DMEM containing 5.5 mM of basal glucose was used to cultivate the cells, and 10% FBS, 100 µg/mL penicillin/streptomycin, and 2 mM of L-glutamine were added to the media. SH-SY5Y was kept in a humidified incubator with 5% CO_2_ at 37 °C. Trypsinization was used to separate cells that reached 80% confluence from the flask’s surface before they were planted at a density of 1 × 10^4^ cells/cm^2^ onto 96-well plates. To assess the impact of glucose dosage on cell viability, additional glucose was added to reach final concentrations of 10, 20, 30, 40, 50, and 100 mM, respectively, and the wells were then incubated for 24 h (each group had three replications). The incubation period was selected based on the findings of earlier studies [31,32]. Cells treated with mannitol (150 mM) were included as an osmotic pressure control group [33]. Following the application of 10 μL of MTT solution to each well, the plate was incubated for four hours at 37 °C. After removal of the MTT solution, DMSO was applied to each well and left for fifteen minutes. Lastly, a microplate reader was used to measure each well’s absorbance at 570 nm.

### 4.3. Transfection of SH-SY5Y with lncRNA MEG3 siRNA

Through use of a Lipofectamine RNAiMAX transfection kit (Thermo Fisher Scientific, Waltham, MA, USA), SH-SY5Y cells were transfected with small interfering RNA (siRNA) targeting *MEG3* (si*MEG3*) and negative control siRNA (siNC) following the manufacturer’s instructions. The normoglycemic and hyperglycemic groups were seeded with 1 × 10^5^ cells into a six-well plate containing Opti-MEM 24 h before transfection. Following incubation, cells were transfected with 30 pmol of NC siRNA and 30 pmol of lncRNA *MEG3* siRNA using Lipofectamine RNAiMax reagent for 24 h at 37 °C. SH-SY5Y cells without the transfection protocol were the control (C) group. The lncRNA *MEG3* knockdown efficiency at the mRNA expression level was evaluated using qRT-PCR assay.

### 4.4. Quantitative Real Time-Polymerase Chain Reaction (qRT-PCR)

Total RNA was extracted from cells using a Trizol reagent (Thermo Fisher Scientific, Waltham, MA, USA). Subsequently, cDNA synthesis was carried out from RNA samples of each group using the High Capacity cDNA Reverse Transcription Kit (Applied Biosystems, Foster City, CA, USA). Following this, the mRNA expression levels were asses using a QuantiTect SYBR Green PCR Kit (Qiagen, Hilden, Germany). Thermo Fisher Scientific Step One Plus Real-Time PCR was used to quantify the relative expression levels of transcripts via 2^−∆∆Ct^ calculation after they were normalized to the endogenous control β-actin. Every sample was replicated three times. Supplementary Table S1 displays the primer sequences utilized in the reactions. The graphs related to qRT-PCR were generated using GraphPad Prism version 8.0.2.

### 4.5. Western Blotting

Protease/phosphatase inhibitors were used to extract the total protein from SH-SY5Y cells in RIPA Lysis Buffer, and the Bradford protein assay was used to determine quantities in accordance with the guidelines provided by the manufacturer. The samples were put onto nitrocellulose membranes after being subjected to 4–15% sodium dodecyl sulfate-polyacrylamide gel electrophoresis (SDS-PAGE), with 50 μg of protein in each well. The membranes were immunoblotted with phosphorylated tau (FineTest, Fnab10924), phosphorylated GSK3β (FineTest, Fnab10504), and ACTB (FineTest, Fnab00869). Afterward, the membranes were probed using HRP-conjugated secondary antibodies (FineTest, FNSA-0004), visualized using a detection system, investigated with the detection system’s Genesis (Gene Tools) program, and quantified using ImageJ software’s densitometry analysis, version 1.53t (NIH, Washington, DC, USA). The signals were detected using an enhanced chemiluminescence (ECL) detection system (Thermo Fisher Scientific). Densitometric analysis of Western blot bands was performed using ImageJ software (NIH, Bethesda, MD, USA). The intensity of each protein band was quantified and normalized to the corresponding loading control (ACTB). Relative protein expression levels were calculated by comparing the normalized values among the experimental groups. Western blot analyses were generated using GraphPad Prism version 8.0.2.

### 4.6. Network Analyses

KEGG pathway enrichment analysis was performed using the STRING v12 database for the terms “Alzheimer’s disease” (term ID: hsa05010), “Type II diabetes mellitus” (term ID: hsa04930), and “Insulin resistance” (term ID: hsa04931). Additionally, term enrichment was conducted using the Disease-Gene Associations (DISEASES) database for Alzheimer’s disease (term ID: DOID:10652), diabetes mellitus (term ID: DOID:9351), and hyperglycemia (term ID: DOID:4195). A protein–protein interaction (PPI) network analysis was performed for the genes commonly identified in both categories, using a minimum required interaction score of 0.7. For the genes identified as upregulated or downregulated based on qPCR and Western blot analyses, biological process (Gene Ontology) enrichment analysis was performed, and the top 10 enriched terms were determined [34]. Protein-coding genes and regulatory RNA molecules associated with *MEG3* (ENSG00000214548) were identified using the ENCORI (The Encyclopedia of RNA Interactomes) STARBASE v3.0 database [35]. The search was conducted by selecting the following RNA biotypes: protein-coding, pseudogene, rRNA, snRNA, lncRNA, and Mt_rRNA. The genome assembly was set to hg38, and the organism selected was human. We chose RNA–RNA and lncRNA–RNA interaction modes and applied a low stringency threshold of ≥1 to support the experiment. To ensure experimental evidence, CLIP-seq supported interactions were selected. *MEG3* was used as the query gene. These networks were visualized using Cytoscape 3.10.1 [36].

### 4.7. Statistics

Depending on whether the data satisfied the assumptions, either one-way ANOVA or the Kruska–Wallis test was employed for comparisons involving more than two groups. The Tukey and Games–Howell tests were employed for multiple comparisons of groups with homogeneous variances and normally distributed data; for non-normally distributed data, the Bonferroni-adjusted Mann–Whitney U test was employed. Boxplots were made using R’s “ggplot2” program. The mean and standard deviation are used to display continuous variables. IBM SPSS Statistics Version 20.0 was utilized for all statistical analyses, and a significance level of 0.05 was chosen.

## 5. Conclusions

This study highlights that lncRNA *MEG3* may serve as a potential marker for mitigating hyperglycemia-induced neurotoxicity and associated pathologies, reflecting the microenvironment observed in Alzheimer’s disease. This suggests that lncRNA *MEG3* may be a promising therapeutic target in the clinical picture of AD, particularly by modulating molecular pathways and inflammatory responses that contribute to disease progression. The significant upregulation of *GSK3β*, *TAU*, *IL1β*, *IL6*, and *TNFα* genes observed in our model aligns with extensive literature implicating these molecules in the pathological mechanisms of AD.

## Data Availability

Data supporting the findings of this study are available within the article. Further queries can be addressed upon reasonable request to the corresponding author.

## Supplementary Materials

The information supporting this article can be downloaded at https://www.mdpi.com/article/10.3390/ijms26167944/s1.

## Abbreviations

The following abbreviations are used in this manuscript:

AD	Alzheimer’s disease
LncRNA MEG3	long non-coding RNA maternally expressed gene 3
T3D	type 3 diabetes
T2D	type 2 diabetes
PI3K	phosphatidylinositol-3-kinase
AKT	protein kinase B
GSK3β	glycogen synthase kinase 3-beta
HG	high glucose
Hyp	hyperglycemic model
siRNA	small interfering RNA
siMEG3	small interfering MEG3
qRT-PCR	quantitative real-time polymerase chain reaction
MAPT, TAU	microtubule-associated protein TAU
IL1β	interleukin 1 beta
IL6	interleukin 6
TNFα	tumor necrosis factor-α
AGEs	advanced glycation end products
ICA	icariin
Aβ 25-35	amyloid beta 25-35
IRS1	insulin receptor substrate 1
hs-CRP	high-sensitivity C-reactive protein
DMEM	Dulbecco’s modified eagle’s medium
FBS	fetal bovine serum
Opti-MEM	reduced serum medium
CSF	cerebrospinal fluid
DMSO	dimethyl sulfoxide
PBS	phosphate-buffered saline
MTT	thiazolyl blue tetrazolium bromide
Malat1	metastasis-associated lung adenocarcinoma transcript 1
ACTB	beta-actin
p-tau	phosphorylated tau
PPI	protein–protein interaction
SDS-PAGE	sodium dodecyl sulfate–polyacrylamide gel electrophoresis
HRP	horseradish peroxidase
