# Supplementary material for: Regulatory Role of lncRNA MEG3 Silencing on PI3K/GSK3β/Tau Pathway in a High-Glucose-Induced Cell Model
Source: Int J Mol Sci. 2025 Aug 18;26(16):7944. doi: 10.3390/ijms26167944 (PMC12386376; doi:10.3390/ijms26167944)
Supplement: Supplementary file 1 [file ijms-26-07944-s001.zip › ijms-3728386-supplementary.pdf]

Supplementary Table S1: The sequence of the primer pairs was used in qRTPCR.

	Forward Primer (5′ -3′)	Reverse Primer (5′ -3′)
MEG3	CTTTTCTGGGGGAATGGGG	CTTTTCTGGGGGAATGGGG
PI3K	AGCTGGTCTTCGTTTCCTGA	GAAACTTTTTCCCACCACGA
Akt	ACTCATTCCAGACCCACGAC	CCGGTACACCACGTTCTTCT
GSK3β	CCGACTAACACCACTGGAAGCT	AGGATGGTAGCCAGAGGTGGAT
MAPT (TAU)	CCAAGTGTGGCTCATTAGGCA	CCAATCTTCGACTGGACTCTGT
IL-1β	CCACAGACCTTCCAGGAGAATG	GTGCAGTTCAGTGATCGTACAGG
IL-6	AGACAGCCACTCACCTCTTCAG	TTCTGCCAGTGCCTCTTTGCTG
TNF-α	CTCTTCTGCCTGCTGCACTTTG	ATGGGCTACAGGCTTGTCACTC
β-actin	AACTGGGACGACATGGAGAA	GAAGGTCTCAAACATGATCTGG

Supplementary Table S2: The identified genes and their associated pathways with lncRNA MEG3 (ENSG00000214548).

GeneID	GeneName	GeneType	interac Num	Exp Num	seqType Num	Total Reads Num	Free Energy	AlignScore
ENSG00000006451	*RALA*	protein_coding	1	1	1	1	-20,10	22,00
ENSG00000011405	*PIK3C2A*	protein_coding	1	1	1	1	-22,20	20,00
ENSG00000031081	*ARHGAP31*	protein_coding	1	1	1	1	-32,70	14,00
ENSG00000048828	*FAM120A*	protein_coding	1	1	1	1	-26,20	10,00
ENSG00000057663	*ATG5*	protein_coding	1	1	1	1	-19,50	10,00
ENSG00000068615	*REEP1*	protein_coding	1	1	1	1	-23,80	15,50
ENSG00000069869	*NEDD4*	protein_coding	1	1	1	1	-19,60	10,50
ENSG00000078304	*PPP2R5C*	protein_coding	1	1	1	2	-60,30	12,00
ENSG00000104231	*ZFAND1*	protein_coding	1	1	1	1	-17,50	13,50
ENSG00000110066	*KMT5B*	protein_coding	1	1	1	1	-22,70	19,50
ENSG00000111669	*TPI1*	protein_coding	1	1	1	1	-19,50	10,00
ENSG00000112159	*MDN1*	protein_coding	1	1	1	1	-21,90	18,00
ENSG00000112335	*SNX3*	protein_coding	1	1	1	1	-15,90	12,00
ENSG00000112541	*PDE10A*	protein_coding	1	1	1	2	-30,20	19,50
ENSG00000112851	*ERBIN*	protein_coding	1	1	1	1	-49,90	20,00
ENSG00000122912	*SLC25A16*	protein_coding	1	1	1	1	-23,60	12,00
ENSG00000125746	*EML2*	protein_coding	1	1	1	1	-17,20	12,00
ENSG00000125901	*MRPS26*	protein_coding	1	1	1	1	-18,40	10,00
ENSG00000130119	*GNL3L*	protein_coding	1	1	1	1	-17,50	12,00
ENSG00000130816	*DNMT1*	protein_coding	1	1	1	2	-37,10	19,50
ENSG00000131374	*TBC1D5*	protein_coding	1	1	1	1	-44,50	12,50
ENSG00000132024	*CC2D1A*	protein_coding	1	1	1	1	-21,20	15,00
ENSG00000137288	*UQCC2*	protein_coding	1	1	1	1	-55,70	25,00
ENSG00000140650	*PMM2*	protein_coding	1	1	1	1	-20,40	20,00
ENSG00000141622	*RNF165*	protein_coding	1	1	1	3	-20,40	13,50
ENSG00000142892	*PIGK*	protein_coding	1	1	1	1	-32,20	26,00
ENSG00000145087	*STXBP5L*	protein_coding	1	1	1	1	-28,90	16,50
ENSG00000146072	*TNFRSF21*	protein_coding	1	1	1	1	-18,60	10,00
ENSG00000150760	*DOCK1*	protein_coding	1	1	1	1	-22,10	17,00
ENSG00000150990	*DHX37*	protein_coding	1	1	1	1	-26,60	12,00
ENSG00000153944	*MSI2*	protein_coding	1	1	1	1	-24,10	10,50
ENSG00000154654	*NCAM2*	protein_coding	1	1	1	1	-18,60	16,00
ENSG00000157445	*CACNA2D3*	protein_coding	1	1	1	2	-45,00	24,00
ENSG00000157985	*AGAP1*	protein_coding	1	1	1	1	-19,30	12,00

ENSG00000162600	*OMA1*	protein_coding	1	1	1	1		-17,70	20,50
ENSG00000164808	*SPIDR*	protein_coding	1	1	1	1		-23,50	11,50
ENSG00000166147	*FBN1*	protein_coding	1	1	1	1		-47,60	20,00
ENSG00000166348	*USP54*	protein_coding	1	1	1	1		-18,50	13,50
ENSG00000168066	*SF1*	protein_coding	1	1	1	1		-48,60	22,00
ENSG00000168137	*SETD5*	protein_coding	1	1	1	1		-38,10	19,00
ENSG00000168702	*LRP1B*	protein_coding	1	1	1	1		-27,30	22,00
ENSG00000168827	*GFM1*	protein_coding	1	1	1	1		-25,40	10,50
ENSG00000170832	*USP32*	protein_coding	1	1	1	1		-30,30	15,50
ENSG00000171943	*SRGAP2C*	protein_coding	1	1	1	2		-23,90	14,00
ENSG00000172572	*PDE3A*	protein_coding	1	1	1	3		-27,50	17,50
ENSG00000174669	*SLC29A2*	protein_coding	1	1	1	1		-41,90	16,00
ENSG00000175582	*RAB6A*	protein_coding	1	1	1	2		-48,90	15,50
ENSG00000185305	*ARL15*	protein_coding	1	1	1	1		-23,50	20,00
ENSG00000196233	*LCOR*	protein_coding	1	1	1	1		-31,20	12,00
ENSG00000197056	*ZMYM1*	protein_coding	1	1	1	1		-35,00	20,00
ENSG00000197302	*ZNF720*	protein_coding	1	1	1	1		-35,90	14,00
ENSG00000197472	*ZNF695*	protein_coding	1	1	1	1		-23,60	17,50
ENSG00000206527	*HACD2*	protein_coding	1	2	1	47		-15,20	22,50
ENSG00000053438	*NNAT*	protein_coding	1	1	1	1		-18,50	24,00
ENSG00000082701	*GSK3B*	protein_coding	1	1	1	1		-27,50	19,00
ENSG00000100139	*MICALL1*	protein_coding	1	1	1	1		-32,00	16,00
ENSG00000109118	*PHF12*	protein_coding	1	1	1	1		-35,10	19,50
ENSG00000112855	*HARS2*	protein_coding	1	1	1	1		-20,90	10,00
ENSG00000113448	*PDE4D*	protein_coding	1	1	1	1		-26,70	16,50
ENSG00000120899	*PTK2B*	protein_coding	1	1	1	1		-18,20	11,50
ENSG00000129250	*KIF1C*	protein_coding	1	1	1	1		-26,70	16,00
ENSG00000136240	*KDELR2*	protein_coding	1	1	1	1		-49,40	20,00
ENSG00000140443	*IGF1R*	protein_coding	1	1	1	1		-20,20	10,00
ENSG00000143341	*HMCN1*	protein_coding	1	1	1	1		-21,60	23,00
ENSG00000145569	*OTULINL*	protein_coding	1	1	1	2		-15,40	12,00
ENSG00000157193	*LRP8*	protein_coding	1	1	1	1		-72,40	35,00
ENSG00000157637	*SLC38A10*	protein_coding	1	1	1	1		-34,90	24,00
ENSG00000158805	*ZNF276*	protein_coding	1	1	1	1		-21,60	17,50
ENSG00000164327	*RICTOR*	protein_coding	1	1	1	1		-34,90	18,50
ENSG00000165650	*PDZD8*	protein_coding	1	1	1	2		-19,20	22,00
ENSG00000167522	*ANKRD11*	protein_coding	1	1	1	1		-70,40	26,00
ENSG00000167548	*KMT2D*	protein_coding	1	1	1	1		-101,90	85,00
ENSG00000171843	*MLLT3*	protein_coding	1	1	1	1		-21,50	16,00
ENSG00000175265	*GOLGA8A*	protein_coding	1	1	1	2		-69,30	22,00
ENSG00000184743	*ATL3*	protein_coding	1	1	1	1		-17,10	14,00
ENSG00000186073	*CDIN1*	protein_coding	1	1	1	2		-31,10	19,50
ENSG00000196498	*NCOR2*	protein_coding	1	1	1	1		-23,60	13,50
ENSG00000198786	*MT-ND5*	protein_coding	1	1	1	2		-24,10	12,50
ENSG00000198899	*MT-ATP6*	protein_coding	1	1	1	2		-41,20	19,00
ENSG00000204839	*MROH6*	protein_coding	1	1	1	1		-75,20	18,50
ENSG00000235194	*PPP1R3E*	protein_coding	1	1	1	1		-31,10	17,50
ENSG00000215203	*GRXCR1*	protein_coding	1	1	1	1		-28,60	18,50
ENSG00000234719	*NPIPB2*	protein_coding	1	1	1	1		-32,60	19,00
ENSG00000285238	AC006064.6	protein_coding	1	1	1	1		-41,20	15,00
PGOHUM00000304129	PGOHUM00000304129	pseudogene	1	1	1	1		-22,70	13,50
LSU-rRNA	LSU-rRNA	rRNA	2	2	2	2		-25,90	23,00
NR_146151_blat	RNA45SN3	rRNA	7	6	2	8		-30,80	20,50
NR_146151.1	RNA45SN3	rRNA	1	1	1	1		-38,20	14,00
SSU-rRNA	SSU-rRNA	rRNA	2	2	1	2		-47,00	22,00
ENSG00000206652	RNU1-1	snRNA	1	1	1	1		-29,10	10,00
ENSG00000270722	RNVU1-31	snRNA	1	1	1	1		-27,60	17,50
ENSG00000204464	C1orf195	lncRNA	1	1	1	1		-32,90	18,50
ENSG00000214188	ST7-OT4	lncRNA	1	1	1	1		-48,60	19,00
ENSG00000232995	RGS5	lncRNA	1	1	1	1		-17,00	12,00
ENSG00000245532	NEAT1	lncRNA	1	1	1	1		-27,70	17,00
ENSG00000255248	MIR100HG	lncRNA	1	1	1	1		-16,00	10,00
ENSG00000258938	AL162311.3	lncRNA	1	1	1	1		-38,60	12,50

ENSG00000214783	POLR2J4	lncRNA	1	1	1	1	-33,60	14,00
ENSG00000233452	STXBP5-AS1	lncRNA	1	1	1	1	-30,00	21,50
ENSG00000251562	MALAT1	lncRNA	3	1	1	5	-38,80	20,00
ENSG00000258399	AL117190.1	lncRNA	1	1	1	2	-36,20	17,50
ENSG00000258663	AL117190.2	lncRNA	1	1	1	1	-47,70	13,50
ENSG00000210082	MT-RNR2	Mt_rRNA	1	1	1	15	-15,40	20,00

Supplementary Table S3: RNA molecules associated with lncRNA MEG3 (ENSG00000214548).

	KEGG			DISEASES		
Genes	Alzheimer disease (hsa0501)	Type II diabetes mellitus (hsa04930)	Insulin resistance (hsa04931)	Alzheimer disease (DOID10652)	Diabetes mellitus (DOID9351)	Hyperglycemia (DOID4195)
A2M				●		
ABCA7				●		
ABCC8		●			●	
ACACB			●			
ACE					●	
ACHE				●		
ADAM10	●			●		
ADAM17	●					
ADIPOQ		●			●	●
ADRB3					●	
ADRM1	●					
AGER	●					
AGPAT2					●	
AGT			●			
AKT1	●		●		●	●
AKT2	●		●		●	●
AKT3	●		●			
ALB					●	●
ALMS1					●	
ANGPTL8					●	
APAF1	●					
APBB1	●					
APC	●					
APC2	●					
APCS				●	●	
APH1A	●					
APH1B	●					
APOE	●			●	●	
APP	●			●		
APPL1					●	
ARAF	●					
ATF4	●					
ATF6	●					
ATG101	●					
ATG14	●					
ATG2A	●					
ATG2B	●					
ATP2A1	●					
ATP2A2	●					
ATP2A3	●					
ATP5F1A	●					
ATP5F1B	●					
ATP5F1C	●					
ATP5F1D	●					
ATP5F1E	●					

ATP5MC1	●				
ATP5MC2	●				
ATP5MC3	●				
ATP5PB	●				
ATP5PD	●				
ATP5PF	●				
ATP5PO	●				
AXIN1	●				
AXIN2	●				
BACE1	●			●	
BACE2	●				
BAD	●				
BCAR1					●
BDNF				●	
BECN1	●				
BECN2	●				
BID	●				
BLK					●
BRAF	●				
BSCL2					●
C11orf24					●
CACNA1A		●			
CACNA1B		●			
CACNA1C	●	●			
CACNA1D	●	●			
CACNA1E		●			
CACNA1F	●				
CACNA1G		●			
CACNA1S	●				
CALM3	●				
CALML3	●				
CALML4	●				
CALML5	●				
CALML6	●				
CAPN1	●				
CAPN10					●
CAPN2	●				
CASP3	●				
CASP7	●				
CASP8	●				
CASP9	●				
CAV1					●
CAVIN1					●
CCR5					●
CD36			●		
CD38					●
CDK5	●				
CDK5R1	●				
CDKAL1					●
CEL					●
CELA2A					●
CHRM1	●				
CHRM3	●				
CHRM5	●				
CHUK	●				
CISD2					●
CLEC16A					●
CLPS					●
CLU				●	
COX4I1	●				
COX4I2	●				
COX5A	●				
COX5B	●				
COX6A1	●				

COX6A2	●				
COX6B1	●				
COX6B2	●				
COX6C	●				
COX7A1	●				
COX7A2	●				
COX7A2L	●				
COX7B	●				
COX7B2	●				
COX7C	●				
COX8A	●				
COX8C	●				
CPT1A		●			
CPT1B		●			
CR1L			●		
CREB1		●			
CREB3		●			
CREB3L1		●			
CREB3L2		●			
CREB3L3		●			
CREB3L4		●			
CREB5		●			
CRP				●	
CRTC2		●			
CSF1	●				
CSNK1A1L	●				
CSNK1E	●				
CSNK2A1	●				
CSNK2A2	●				
CSNK2A3	●				
CSNK2B	●				
CST3			●		
CTLA4				●	
CTNNB1	●				
CTSB			●		
CTSD			●		
CYBB	●				
CYC1	●				
CYCS	●				
DBN1			●		
DCAF17				●	
DDIT3	●				
DEFB105B				●	
DKK1	●				
DKK2	●				
DKK4	●				
DMXL2				●	
DNAJC3				●	
DPP4				●	●
DVL1	●				
DVL2	●				
DVL3	●				
DYRK1B				●	
EIF2AK2	●				
EIF2AK3	●			●	
EIF2S1	●				
ENPP1				●	
ERN1	●				
EXOC3L2			●		
FADD	●				
FAS	●				
FDX1			●		
FOXO1		●			
FOXP3				●	

FRAT1	●					
FRAT2	●					
FZD1	●					
FZD10	●					
FZD2	●					
FZD3	●					
FZD4	●					
FZD5	●					
FZD6	●					
FZD7	●					
FZD8	●					
FZD9	●					
G6PC			●			
G6PC2			●			
G6PC3			●			
GAD2					●	
GAPDH	●					
GCG					●	●
GCGR					●	
GCK		●			●	
GFPT1			●			
GFPT2			●			
GLIS3					●	
GLP1R					●	●
GNAQ	●					
GPR83	●					
GRIN1	●					
GRIN2A	●					
GRIN2B	●					
GRIN2C	●					
GRIN2D	●					
GRM5	●					
GSK3A				●	●	
GSK3B	●		●	●	●	
GYS1			●		●	
GYS2			●			
HBA1					●	
HK1		●				
HK2		●				
HK3		●				
HKDC1		●				
HLA-DQA1					●	
HLA-DQB1					●	
HLA-DRB1					●	
HNF1A					●	
HNF1B					●	
HNF4A					●	
HRAS	●					
HSD17B10	●					
HSPG2				●	●	
IAPP					●	
IDE	●			●		
IER3IP1					●	
IFIH1					●	
IGF1					●	
IGF2BP2					●	
IKBKB	●	●	●			
IL1A	●					
IL1B	●				●	
IL2RA					●	
IL6	●		●	●	●	●
INPPL1					●	
INS	●	●	●		●	●
INSR	●	●	●		●	

IRS1	●	●	●		●	●
IRS2	●	●	●			
IRS4	●	●				
ITPR1	●					
ITPR2	●					
ITPR3	●				●	
KCNJ11		●			●	
KCNQ1					●	
KIF5A	●					
KIF5B	●					
KIF5C	●					
KLC1	●					
KLC2	●					
KLC3	●					
KLC4	●					
KLF11					●	
KRAS	●					
LEP					●	●
LIPE					●	
LPL	●					
LRP1	●					
LRP5	●					
LRP6	●					
MAFA		●			●	
MAP2K1	●					
MAP2K2	●					
MAP2K7	●					
MAP3K5	●					
MAPK1	●	●				
MAPK3	●	●				
MAPK8	●	●	●			
MAPK8IP1					●	
MAPK9	●	●	●			
MAPT	●			●		
MCF2L2					●	
MCU	●					
MIA3					●	
MINK1				●		
MLX			●			
MLXIP			●			
MLXIPL			●			
MME	●					
MSANTD4				●		
MT-ATP6	●					
MT-ATP8	●					
MT-CO1	●					
MT-CO2	●					
MT-CO3	●					
MT-CYB	●					
MT-ND1	●			●	●	
MT-ND2	●			●		
MT-ND3	●					
MT-ND4	●					
MT-ND4L	●					
MT-ND5	●					
MT-ND6	●					
MTNR1B					●	
MTOR	●	●	●			
NAE1	●					
NCSTN	●					
NDUFA1	●					
NDUFA10	●					
NDUFA11	●					
NDUFA12	●					

NDUFA13	●				
NDUFA2	●				
NDUFA3	●				
NDUFA4	●				
NDUFA4L2	●				
NDUFA5	●				
NDUFA6	●				
NDUFA7	●				
NDUFA8	●				
NDUFA9	●				
NDUFAB1	●				
NDUFB1	●				
NDUFB10	●				
NDUFB2	●				
NDUFB3	●				
NDUFB4	●				
NDUFB5	●				
NDUFB6	●				
NDUFB7	●				
NDUFB8	●				
NDUFB9	●				
NDUFC1	●				
NDUFC2	●				
NDUFS1	●				
NDUFS2	●				
NDUFS3	●				
NDUFS4	●				
NDUFS5	●				
NDUFS6	●				
NDUFS7	●				
NDUFS8	●				
NDUFV1	●				
NDUFV2	●				
NDUFV3	●				
NEUROD1					●
NFKB1	●		●		
NFKBIA			●		
NOS1	●				
NOS2	●				
NOS3			●		
NOX1	●				
NOX4	●				
NR1H2			●		
NR1H3			●		
NRAS	●				
NRBF2	●				
NTAN1					●
OAS1					●
OGA			●		
OGT			●		
PALD1				●	
PAX4					●
PCK1			●		
PCK2			●		
PDPK1			●		
PDX1		●			●
PIK3C3	●				
PIK3CA	●	●	●		
PIK3CB	●	●	●		
PIK3CD	●	●	●		
PIK3R1	●	●	●		
PIK3R2	●	●	●		
PIK3R3	●	●	●		
PIK3R4	●				

PKLR		●				
PKM		●				
PLAGL1					●	
PLCB1	●					
PLCB2	●					
PLCB3	●					
PLCB4	●					
PMP22					●	
PPARA			●		●	
PPARG					●	●
PPARGC1A			●		●	
PPARGC1B			●			
PPID	●					
PPIF	●					
PPP1CA			●			
PPP1CB			●			
PPP1CC			●			
PPP1R15B					●	
PPP1R3A			●		●	
PPP1R3B			●			
PPP1R3C			●			
PPP1R3D			●			
PPP1R3E			●			
PPP3CA	●					
PPP3CB	●					
PPP3CC	●					
PPP3R1	●					
PPP3R2	●					
PPP5C				●		
PRKAA1			●			
PRKAA2			●			
PRKAB1			●			
PRKAB2			●			
PRKAG1			●			
PRKAG2			●			
PRKAG3			●			
PRKCB			●			
PRKCD		●	●			
PRKCE		●	●			
PRKCQ			●			
PRKCZ		●	●			
PRRT1				●		
PSEN1	●			●		
PSEN2	●			●		
PSENEN	●					
PSMA1	●					
PSMA2	●					
PSMA3	●					
PSMA4	●					
PSMA5	●					
PSMA6	●					
PSMA7	●					
PSMA8	●					
PSMB1	●					
PSMB2	●					
PSMB3	●					
PSMB4	●					
PSMB5	●					
PSMB6	●					
PSMB7	●					
PSMC1	●					
PSMC2	●					
PSMC3	●					
PSMC4	●					

PSMC5	●					
PSMC6	●					
PSMD1	●					
PSMD11	●					
PSMD12	●					
PSMD14	●					
PSMD2	●					
PSMD3	●					
PSMD4	●					
PSMD7	●					
PSMD8	●					
PSMD9	●					
PTEN			●			
PTF1A					●	
PTGS2	●					
PTPN1			●			
PTPN11			●			
PTPN22					●	
PTPRF			●			
PTPRN					●	
PTPRN2					●	
PYGB			●			
PYGL			●			
PYGM			●			
RAF1	●					
RALGPS2				●		
RB1CC1	●					
RELA	●		●			
RETN					●	
RETNLB					●	
RFX6					●	
RPS6KA1			●			
RPS6KA2			●			
RPS6KA3			●			
RPS6KA6			●			
RPS6KB1			●			
RPS6KB2			●			
RTN3	●					
RTN4	●					
RYR3	●					
SDHA	●					
SDHB	●					
SDHC	●					
SDHD	●					
SEL1L					●	
SERPINA3				●		
SH2B3					●	
SLC16A11					●	
SLC16A13					●	
SLC19A2					●	
SLC22A3					●	
SLC25A31	●					
SLC25A4	●					
SLC25A5	●					
SLC25A6	●					
SLC27A1			●			
SLC27A2			●			
SLC27A3			●			
SLC27A4			●			
SLC27A5			●			
SLC27A6			●			
SLC2A1			●			
SLC2A2		●	●			●
SLC2A4		●	●		●	●

SLC30A10					●	
SLC30A8					●	
SLC5A2					●	●
SNCA	●			●		
SOCS1		●				
SOCS2		●				
SOCS3		●	●			
SOCS4		●				
SORL1				●		
SREBF1			●			
STAT3			●		●	
SUMO4					●	
TBC1D4			●		●	
TCF7L2					●	
TECPR2				●		
TIAF1				●		
TMTC1					●	
TNF	●	●	●		●	
TNFRSF1A	●		●			
TOMM40				●		
TRAF2	●					
TREM2				●		
TRIB3			●			
TRMT10A					●	
TUBA1A	●					
TUBA1B	●					
TUBA1C	●					
TUBA3D	●					
TUBA3E	●					
TUBA4A	●					
TUBA8	●					
TUBAL3	●					
TUBB	●					
TUBB1	●					
TUBB2A	●					
TUBB2B	●					
TUBB3	●					
TUBB4A	●					
TUBB4B	●					
TUBB6	●					
TUBB8	●					
UCP1					●	
UCP3					●	
ULK1	●					
ULK2	●					
UNC5C				●		
UQCR10	●					
UQCR11	●					
UQCRB	●					
UQCRC1	●					
UQCRC2	●					
UQCRFS1	●					
UQCRH	●					
UQCRHL	●					
UQCRQ	●					
VDAC1	●					
VDAC2	●					
VDAC3	●					
VWA2					●	
WFS1					●	
WIPI1	●					
WIPI2	●					
WNT1	●					
WNT10A	●					

WNT10B	●	
WNT11	●	
WNT16	●	
WNT2	●	
WNT2B	●	
WNT3	●	
WNT3A	●	
WNT4	●	
WNT5A	●	
WNT5B	●	
WNT6	●	
WNT7A	●	
WNT7B	●	
WNT8A	●	
WNT8B	●	
WNT9A	●	
WNT9B	●	
XBP1	●	
YIPF5		●
ZFP57		●
